# Socioeconomic inequality of diabetes patients’ health care utilization in Denmark

**DOI:** 10.1186/s13561-017-0155-5

**Published:** 2017-05-26

**Authors:** Camilla Sortsø, Jørgen Lauridsen, Martha Emneus, Anders Green, Peter Bjødstrup Jensen

**Affiliations:** 1 Centre of Health Economics Research (COHERE), Department of Business and Economics, University of Southern Denmark, Campusvej 55, DK-5s30 Odense M, Denmark; 2 Institute of Applied Economics and Health Research (ApEHR), Copenhagen, Denmark; 3 Odense Patient data Explorative Network (OPEN), Odense University Hospital and University of Southern Denmark, Copenhagen, Denmark

**Keywords:** Health inequality, Diabetes, Health care service usage: decomposition, Socio-economic inequality, I10, I12, I14, I18

## Abstract

Understanding socioeconomic inequalities in health care is critical for achieving health equity. The aim of this paper is threefold: 1) to quantify inequality in diabetes health care service utilization; 2) to understand determinants of these inequalities in relation to socio-demographic and clinical morbidity factors; and 3) to compare the empirical outcome of using income level and educational level as proxies for Socio Economic Status (SES).

Data on the entire Danish population of diabetes patients in 2011 (*N* = 318,729) were applied. Patients’ unique personal identification number enabled individual patient data from several national registers to be linked. A concentration index approach with decomposition into contributing factors was applied. Differences in diabetes patients’ health care utilization patterns suggest that use of services differ among patients of lower and higher SES, despite the Danish universal health care system. Especially, out-patient services, rehabilitation and specialists in primary care show different utilization patterns according to SES. Comparison of the empirical outcome from using educational level and income level as proxy for patients’ SES indicate important differences in inequality estimates. While income, alike other measures of labor market attachment, to a certain extent is explained by morbidity and thus endogenous, education is more decisive for patients’ ability to take advantage of the more specialized services provided in a universal health care system.

## Background

Persistent differences in health by socio-economic status (SES) have long been a serious health policy concern in many European countries [9, 10, 43]. Evidence on the contributing factors to inequality in health in general and disease specific inequality may guide future efforts to reduce unequal distributions of for instance health care [9, 10]. This study presents – to our knowledge - first time evidence on the composition of socioeconomic inequality in diabetes patients’ health care utilization patterns.

Diabetes Mellitus is one of contemporary time’s most burdensome chronic diseases [52]. It is well known that socio-economic inequality exists in diabetes with higher incidence and mortality among lower socio-economic groups [1, 4, 18, 25, 31, 37]. Despite universal coverage health care systems, social inequalities have been evidenced in most European countries [43]. Several Danish reports have underlined that large differences exist in compliance to treatment, especially preventive efforts and retention of life style changes among chronic patients [11–13]. Access to health care, hence, is not only a question of equal potential access, as in a universal health care system like the Danish. The concept of “realized access” [20] reflects patients’ actual use of the available services. In health care systems with universal coverage, realized access may be constrained by financial and organizational barriers to the use of benefits, such as required co-payments or other out-of-pocket payments, restrictions on specialty referrals, or lack of proximity to health care facilities [20]. Differences in use of health care within patient groups of same need provide insight into patients’ ability to take advantage of the services provided in a universal health care system. Such knowledge can guide future effort to increase success of early detection, secondary prevention and treatment. This is highly important, not only for patients’ quality and length of life, but also for societies to control the costs of the increasing diabetes populations [21, 38].

Several studies have assessed the level of socioeconomic inequalities in health using concentration indices and concentration curves [33, 43, 44, 47]. Taking advantage of the detailed Danish registers, we apply data on individual patient level on all Danish diabetes patients [41]. We have previously documented that diabetes patients of lower SES experience higher morbidity and mortality [39, 40]. Access to comprehensive data on patients’ morbidity patterns is unique, allowing for investigation of novel associations between SES, morbidity and health care utilization patterns investigating the inquiry whether patients’ health care usage reflects their need defined through morbidity.

The present study sets out three research inquiries 1) to quantify socioeconomic inequality in diabetes health care and pharmaceutical usage (reflected through cost accounts), 2) to decompose these inequalities by quantifying the contribution attributable to individual demographic determinants and individual morbidity characteristics, and 3) to compare the empirical outcome from using educational level versus income level as proxy for patients’ SES. Thus, the outline of the paper is as follows. Next to this introduction, Section 2 briefly presents the econometric methods applied. Following this, Section 3 describes data collection and preparation, while the results are presented in Section 4. Finally, Section 5 provides a discussion of the results, while Section 6 rounds off with concluding remarks.

## Methods

Similar to previous studies initiated by Wagstaff *et al*. [49] we use the concentration index as our measure of relative socioeconomic inequality in healthcare costs. A concentration curve *L*(*s*) plots the cumulative proportion of the population (ranked by socioeconomic status (SES), beginning with lowest SES) against the cumulative proportion of costs. If *L*(*s*) coincides with the diagonal everyone is equally off. However, if *L*(*s*) lies below the diagonal, then inequality in healthcare costs exists and favors those with high SES. The further *L*(*s*) lies from the diagonal, the larger the degree of inequality. The concentration index, *C*, is defined as twice the area between *L*(*s*) and the diagonal and takes a value of 0, when everyone is equally off regardless of SES. The minimum and maximum values of *C* are −1 and +1, respectively; these occur in the (hypothetical) situation where costs are concentrated in the hand of the least disadvantaged and the most disadvantaged person, respectively. Thus, the larger negative value of *C*, the more costs concentrate among low SES groups. A computational formula for *C* was given by Kakwani et al. [27] as $C=\frac{2}{ N\mu}{\displaystyle \sum_{i=1}^N{y}_i{R}_i-1}$, where $\mu =\frac{1}{N}{\displaystyle \sum_{i=1}^N{y}_i}$ is the mean of observed costs, *N* the sample size, *y*_*i*_ observed costs, and *R*_*i*_ the fractional rank defined according to Kakwani et al. as ${R}_i=\frac{i-1}{N}+\frac{1}{2}$. Following the same authors, *C* can be conveniently computed as the covariance of *y*_*i*_ and *R*_*i*_, i.e. $C=\frac{2}{\mu}{\operatorname{cov}}_w\left({y}_i,{R}_i\right)=\frac{2}{ N\mu}{\displaystyle \sum_{i=1}^N\left({y}_i-\mu \right)\left({R}_i-\frac{1}{2}\right)}$.

A straightforward way of decomposing the predicted degree of inequality into the contributions of explanatory factors was proposed by Wagstaff et al. [48]. Adapting their approach to the present case, we use a traditional linear regression that links healthcare costs to the determinants, leading to a decomposition of the concentration index of predicted costs as $\widehat{C}={\displaystyle \sum_k\frac{\beta_k{\overline{x}}_k}{\widehat{\mu}}}{C}_k$, where $\widehat{\mu}$ is the mean of predicted costs, ${\overline{x}}_k$ the mean of the determinant *x*_*k*_, and *C*_*k*_ the concentration index of *x*_*k*_ (defined analogously to *C*).

In order to assess sampling variability and to obtain standard errors for the estimated quantities, in particular for the concentration indices and the contributions, i.e. the $\frac{\beta_k{\overline{x}}_k}{\widehat{\mu}}{C}_k$ parts, we apply a “bootstrap” procedure ([14];) in a four-step manner much similar to van Doorslaer et al. [44]: First, a random sub-sample of the size of the original sample is drawn with replacement. Second, the entire set of calculations, as specified above, are performed on this sample. Third, this whole process is repeated 1,000 times, each leading to replicate estimates. Fourth, using the obtained 1,000 replicates, standard deviations and *t* statistics can be computed for all calculated quantities.

## Data

The study is part of the Diabetes Impact Study 2013 [21, 22, 38–40]. Data was collected from the Danish National Diabetes Register (NDR) [6], the Danish National Patient Register [34], the Danish National Prescription Registry [29], the Danish National Health Service Register [3], the Danish Civil Registration System [5], and social registers at Statistics Denmark (SD).

The study population covers all patients registered in NDR diagnosed before 1^st^ of January 2012 and alive 1^st^ of January 2011, described in detail elsewhere (1), leaving *N =* 318,729 patients. The analytical time is a window of one calendar year (2011) in a cross-sectional design. This design does not by definition allow for causational conclusions over time to be drawn, but it enables identification of differences between groups and hence cost pattern exploration [26].

The variables for the study may conveniently be grouped in three overall groups: Patients SES and demographic characteristics; health care usage, and patients’ need for health care.

**Patients’ socioeconomic, demographic and morbidity characteristics** are summarized and described in details in Table 1. Patients’ *annual gross income* is applied as ranking variable, since this measure is the most common measure of SES in the literature analysing inequality through concentration indices [43–45]. Several studies, including those mentioned here, use household rather than individual income, given that the former is a more comprehensive expression of the patients’ economic abilities. Household information, including household income, was not included in the present study. Furthermore, patients’ *highest attained educational level*, based on the Danish Educational Nomenclatura with 13 educational groups, is applied as ranking variable as well, since this measure is frequently used in public health literature due to its simplicity and universality [19]. When education is used as a ranking variable, the full nine level definition is used, and otherwise a three level definition. Following what has been used in previous studies, included demographic variables are *age, gender, ethnicity* (*Dane*, *immigrant* or *descendant*)*, civil status* (*married/partnership, unmarried, widowed/longest living partner* or *divorced/separated*)*, region of residence*, *degree of urbanity of residence*, and *labour market status* (*unemployed*, *early retired*, *retired*, *not in job for other reasons*). Finally, relevant and available morbidity characteristics were added, including *incidence in 2011*, *complication group (CG0, CG1* or *CG2)* and *mortality* (alive or not in 2011) measures. Alike what has been discussed elsewhere, there may be endogeneity problems connected to in particular labour market status, as this affects not only health but also income rank. However, we expect the endogeneity problems to be less profound when using education as SES measure, given that education is taken relatively early in the life course and thus precedes present labour market status.

**Table 1:** Definition of socioeconomic, sociodemographic and morbidity characteristics

Characteristics	Definitions	Categories
Socioeconomics		
Highest educational level attained	Highest educational level attained at date of data extraction, based on the main groups in the Danish educational Nomenclature with 13 educational groups based on years of education.	Variable with 3 or 9 categories: 1) Primary education (<11 years) 2) Middle high education (11 to 15 years 3) Higher education (16+ years) 1) Primary education 2) Upper secondary education 3) Vocational education and training 4) Qualifying educational programmes 5) Short cycle higher education 6) Vocational bachelor’s education 7) Bachelor programmes 8) Master programmes 9) PhD programmes
Income level	Annual gross income 2011 (DKK)	
Demographics		
Gender	Gender	1) Male 2) Female
Age	Age in mid-year	Continuous
Civil status	Marital status	1) Married or in civil partnership 2) Unmarried 3) Widow or longest living partner 4) Divorced or cancelled partnership
Ethnicity	Based on registrations in the Central Person Register 2011.	1) Ethnic Dane 2) Immigrant 3) Descendant
Region of residence	Residence 2011 in relation to the five Danish regions	1) ”Capital Region of Denmark” 2) ”Region Zealand” 3) ”Region of Southern Denmark” 4) “Central Denmark Region” 5) “North Denmark Region”
Urbanity	Residence in type of geographic area in relation to urbanity	1) City 2) Suburbs 3) Outer areas/country side
Occupational status	Affiliation to the labour market	1) Affiliated to the labour market (employed or self-employed) 2) Unemployed (maternal leave, job seeker allowance) 3) Unemployed (unemployment benefit) 4) Education 5) Early retirement 6) Retired 7) Child
Morbidity		
Incident 2011	Patient diagnosed in calendar year 2011	0) Diagnosed in year ≠ 2011 1) Diagnosed in 2011
Complication group at present	Complication group at 31^st^ of December 2011	1) CG0 2) CG1 3) CG2
Complication group at diagnosis	Complication group at diagnosis	1) CG0 2) CG1 3) CG2
Mortality 2011	Death in 2011	0) Alive 2011 1) Death 2011

Turning to **health care usage**, overall volume of treatment related health care, including pharmaceuticals, received by the individual patient, are approximated by the costs of these services through hospital and health insurance statements. This implies that number or type of services is not considered but merely total costs by sectors. Measurements of health care and pharmaceutical consumption in the categories defined, as well as choices of appropriate cost units, are described in details in Table 2. Specifically, a total of nine health care cost components were available, made up of three components summarizing hospital inpatient care (*total inpatient services, inpatient services for stays longer than the average patient in the Diagnosis Related Grouping (DRG) group*, and *inpatient services for rehabilitation*), three components summarizing outpatient care (*total outpatient services, outpatient services for stays longer than the average patient in the DRG group*, and *outpatient services for rehabilitation*), two components summarizing primary care (*services in general practices* and *services for privately practising specialists*), and one component summarizing *prescribed pharmaceutical consumption*.

**Table 2:** Definition of nine health care usage cost components

Cost component	Cost unit
Inpatient and outpatient services delivered in Danish hospitals registered in the National Patient Register divided into the following components: 1) Inpatient services 2) Inpatient services for stays longer than the average patient in this DRG-group 3) Inpatient services for rehabilitation 4) Outpatient services 5) Outpatient services for stays longer than the average patient in this DAGS-group 6) Outpatient services for rehabilitation	Diagnosis Related Grouping (DRG) system and Danish Ambulant Grouping System (DAGS) tariffs - year 2012 [35]. The DRG-tariff system is developed for administrative purpose and based on rough average costs across hospitals for specific diagnostic groups. Excludes interest and depreciation of buildings and equipment while other overhead costs are included
Primary care services delivered by general practitioners and privately practicing specialists such as: dentists, physiotherapists, chiropractors, chiropodists who are registered in the National Health Service Register divided into the following components: 1) Services in general practices 2) Services for privately practicing specialists	Reimbursement fees between the National Health Insurance scheme and private practicing physicians are used as cost units. General Practitioners are compensated by regions through a combination of per capita fee (app. 30% of total) and fee for service (app. 70%) [32]. To reflect this payment scheme in the unit cost, 43.8% of the fee for service in general practice was added on top. Overhead costs covered by capitation fee were hence not distributed across numbers of visits, as would have been most appropriate, but by resource burden.
Prescribed pharmaceuticals dispensed by Danish pharmacies and registered in the Danish national prescription register. (Pharmaceuticals consumed in hospitals are included in DRG-tariffs. Over-the-counter drugs are not included in the statements).	Total sales price includes patient out of pocket payments since costs of prescribed pharmaceuticals are shared between the patient and the primary health care sector by a copayment scheme where patients are reimbursed according to their need. These costs were aggregated since total costs are measured regardless of who pays. 20% VAT was subtracted.

**Patients’ need for health care** should ideally be measured by health care professionals’ clinical assessment of the individual patient. Unfortunately, such data are unavailable, and instead we apply clinically defined morbidity patterns to proxy patients’ need. Patients are classified into three complication groups (*complication group 0 – CG0, complication group 1 – CG1*, and *complication group 2 – CG*2), according to the progression of their diabetes. While CG0 indicates patients without registered complications, CG1 covers those with moderate or minor problems, and CG2 those with severe complications; see Table 3 and Green et al. [21] for details.

**Table 3:** Patient need for health care, as defined by complication state classification

Complication group	Health state^a^
Complication group 0 (CG0)	Diabetes without registered complications
Complication group 1 (CG1)	Moderate or minor complications, problems with eyes, heart, kidney and nervous system, minor amputations below the ancle, bypass operation and some eye operations
Complication group 2 (CG2) –	Severe complications: blindness, amputation above the ancle, severe heart failure, kidney transplant or dialysis

^a^ICD codes defined for each complication group is given in Table 6 in Appendix

## Results

Table 4 presents concentration indices using income as ranking variable. Contributions of socioeconomic, demographic and morbidity determinants to the predicted inequality (the former in percentage of the latter) is presented as well. As income is used as ranking variable, education serves as a control variable only, and thus is applied in the simpler three categories version. Regression coefficients and concentration indices for each of the determinants are given in Table 7 in Appendix.

**Table 4:** Decomposition of inequality in health care and pharmaceutical usage ranked by income

Ranked by income	SECONDARY CARE										PRIMARY CARE				PHARMACEUTICALS	
	INPATIENT						OUTPATIENT									
	Care		long stays		Rehabilitation		Care		Rehabilitation		General pracitice		Specialist			
Number of observations	318,684		318,684		318,684		209,530		8,089		318,684		318,684		318,684	
Mean	20996.5	***	718.561	***	236.955	***	16836.740	***	3954.549	***	3069.671	***	2108.611	***	7489.03	***
C (observed)	−0.176	***	−0.230	***	−0.202	***	−0.036	***	0.024	***	−0.063	***	0.003	**	−0.038	***
C (predicted)	−0.180	***	−0.245	***	−0.207	***	−0.043	***	0.011	*	−0.064	***	−0.026	***	−0.040	***
C (unexplained)	0.004	*	0.016	**	0.004		0.006	***	0.013	***	0.001		0.029	***	0.002	**
Determinants (reference group)	C	Sig.	C	Sig.	C	Sig.	C	Sig.	C	Sig.	C	Sig.	C	Sig.	C	Sig.
SES																
Income	0.06		−0.81		0.28		−3.41		−3.72		1.10	*	99.38		−2.81	
Educational level (high education)																
Primary education	3.67	***	2.73		0.81		−14.15	***	29.22		14.78	***	97.09		2.46	
Medium education	−1.21	***	−1.18		0.39		2.26		−5.93		−4.89	***	1.99		2.45	***
Age and gender (Males0-14) (Females0-14)																
M15-29	−0.66	***	−0.86	***	0.18	**	−3.55	***	2.49		0.16	***	1.88		−0.82	***
M30-44	6.73	***	6.29	***	−1.76	**	24.22	***	−25.07		−6.20	***	−11.23		9.30	***
M45-59	20.45	***	20.20	***	−9.69	***	71.29	***	−58.47		−32.51	***	28.93		49.04	***
M60-74	1.80	***	1.97	***	−1.09	***	3.93	***	−8.11		−3.93	***	7.23		5.58	***
M75+	−10.65	***	−9.91	***	9.75	***	−45.51	***	40.92		26.83	***	−36.67		−33.88	***
F15-29	−0.53	***	−0.55	***	0.16	**	−1.74	***	2.06		1.05	***	−4.84		−1.25	***
F30-44	5.83	***	5.69	***	−2.43	***	17.72	***	−24.96		−10.59	***	54.68		17.01	***
F45-59	22.60	***	19.94	***	−10.61	***	78.07	***	−98.23		−42.88	***	205.85		50.83	***
F60-74	2.18	***	2.04	***	−1.25	***	4.53	***	−17.74		−4.14	***	17.25		4.66	***
F75+	−13.21	***	−11.54	***	10.23	***	−50.92	***	47.46		24.59	***	−49.24		−27.46	***
Labour market affiliation (in job)																
Not in job (maternity leave, job seeker allowance)	−0.29	***	−0.36	***	−0.30	***	−0.84	***	4.34		−0.69	***	2.88		−0.10	
Not in job (unemployment benefit)	1.86	***	0.54		0.84	***	2.68	***	−4.15		4.40	***	−21.52		2.81	***
Education, training	−0.63	**	−1.34	***	0.40	***	1.98	**	5.89		−0.87	***	4.43		1.60	*
Early retired	5.95	***	5.47	***	2.98	***	23.32	***	0.74		8.56	***	−114.36		40.97	***
Retired	22.60	***	18.74	***	14.34	***	74.14	***	−63.97		33.23	***	−349.57		71.38	***
Child	−1.18	***	−2.30	***	0.91	***	2.30		7.16		0.73	***	14.16		−3.47	***
Marital status (married)																
Unmarried	0.10	*	−0.35	**	−0.07		0.36	**	1.29		0.25	***	−4.49		−0.75	***
widowed/longest living partner	0.22		0.99	**	0.88	***	−1.91	***	−0.71		−0.11		20.81		0.71	***
Divorced/cancelled partnership	−0.29	***	−0.48	***	−0.40	***	0.78	**	−1.53		0.00		−5.02		−1.20	***
Ethnicity (Ethic Dane)																
Immigrant	−2.37	***	−2.09	***	−2.07	***	−5.93	***	−10.37		0.79	***	35.59		−13.87	***
Descendant	−0.05		−0.13	***	−0.05	**	−0.17		0.09		0.01		0.93		−0.31	***
Region of residence (Capital Region of Denmark)																
Region Zealand	0.02		−0.08		0.00		−0.13		4.30		0.04		3.82		−0.03	
Region of Southern Denmark	−0.64	***	−1.55	***	2.58	***	1.84	***	68.74		1.57	***	34.67		−0.76	***
Central Denmark Region	0.03		0.11		−0.15		−0.01		−36.14		−0.16		1.31		0.05	
North Denmark Region	−0.28	***	−0.35	***	0.64	***	−1.43	***	0.15		1.10	***	21.02		0.08	
Degree of urbanity of residence (cities)																
Suburbs	−0.01		0.02		0.00		−0.05		1.16		0.06	*	1.03		0.07	*
Country side	−0.35	**	−0.02		−0.79	***	−3.56	***	−16.87		1.92	***	25.08		0.46	*
Morbidity indicators																
Incident in 2011	0.18	***	0.01		0.23	***	−0.40	***	3.18		2.55	***	−26.42		3.42	***
Complication group CG1 (CG0) *	−0.67	***	−0.63	***	−0.45	***	−3.90	***	17.93		−0.23	***	2.67		−3.53	***
Complication group CG2 (CG0)*	26.30	***	20.01	***	23.68	***	57.50	***	−69.18		8.06	***	−116.38		50.73	***
Death in 2011	59.76	***	77.45	***	37.84	***	34.38	***	15.37		−8.04	***	152.06		−20.36	***

*CG0 = no complications, CG1 = minor complications, CG2 = severe complications

**Fig. 1:**
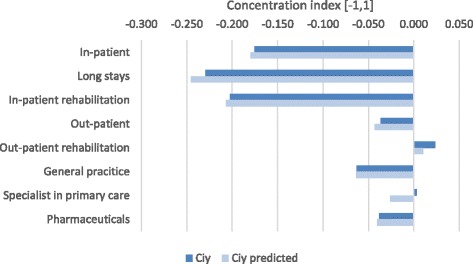
Concentration index (observed and predicted by determinants) of income-related inequalities in cost outcomes Legend: Ciy = Observed concentration index for the outcome variable Ciy predicted = Concentration index predicted by the included determinants for the outcome variable

Overall, the magnitudes of the figures in the table are modest, reflecting the Danish universal health care system with equal access to treatment [50]. Observed and predicted concentration indices for most of the cost variables are negative meaning that costs concentrate among patients of lower income groups. This is illustrated in Fig. 1, where concentration indices to the left are interpreted as costs accumulating among lower SES groups, while the right-side contributions are interpreted reversely.

**Fig. 2:**
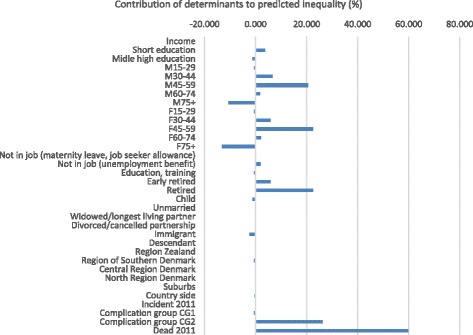
Decomposition of income-related inequality in in-patient care costs

In the decomposition analysis, we included patients’ morbidity patterns, degree of complications at time of analysis, and whether the patient was diagnosed or died in the current year (2011). Ideally, patients’ morbidity patterns should explain inequality in the distribution of health care costs, if costs were allocated exactly according to patients’ need. This, of course, is an unrealistic expectation, since morbidity indicators cannot capture patients’ exact need, and since costs of services cannot proxy the exact received number of services needed. From Fig. 1 it is clear that especially in-patient health care services inhibit inequality, favoring patients with lower incomes. This corresponds well to these patients experiencing higher morbidity and mortality [39, 40]. Looking at the decomposition of inequality for in-patient care, (Fig. 2), it is seen that morbidity patterns explain a large part of predicted inequality. Especially, the morbidity indicators severe complications at time of analysis and death in 2011 have marked influences on inequality in that costs accumulate among patients with these morbidity characteristics, who are also those with the lowest educational level. Among immigrants and elder (75+), the pattern, however, is opposite with costs accumulating to a higher extent among the higher income groups.

As shown in Table 4, concentration indices for outpatient rehabilitation and specialist treatment in primary care are positive, contrary to the other cost variables. However, contributions from determinants are not significant and thus not illustrated.

Turning to the socioeconomic determinants, especially the higher income patients are receiving outpatient services, whereas the lower income patients are receiving more inpatient services and services in general practice.

According to patients’ ethnicity, negative regression coefficients (Table 7 in Appendix), imply that immigrants accumulate lower costs than do ethnic Danes. Given that immigrants have lower incomes (as shown by the negative concentration indices of Table 7 in Appendix), this observation conflicts with the general observation of costs being concentrated among low income groups. However, a potential explanation may be that costs are relatively more concentrated among the higher socioeconomic groups of immigrants than is the case for ethnic Danes. This somewhat surprising tendency, which is observed for in-patient as well as out-patient care and for pharmaceuticals, even when all other demographics and morbidity patterns are taken into account, may be explained by immigrants experiencing language and cultural barriers hindering them in taking full advantage of the Danish universal health care system [12].

For labor market affiliation, the pattern is much similar across cost variables. Especially, being retired contributes highly to the level of inequality with magnitudes around 20-25% of the predicted inequalities in costs. Only children and patients under education have lower costs than patients in job whereas all the other categories in general incur higher costs, especially early retired. Turning to age and gender, it can be seen that these also contribute markedly to inequality. Given that young people are of better health, it is not surprising that they generate lower costs, and it is also to be expected that they have lower incomes, as many of them are studying or in the beginning of their labor market career. However, for the elder group, a potential interpretation may be that elder with low incomes are disfavored with respect to treatment cost.

From differences across the regions, a pattern is seen, which is also reflected in the level of urbanity, where especially residents in the country side use less resources than patients resident in cities, and where costs are more concentrated among patients from higher income levels.

Turning to marital status, divorced patients generally have better income, as indicated by the positive concentration index (Table 7 in Appendix), and they accumulate more in-patient services but less pharmaceutical costs and general practice costs compared to married. The latter corresponds well with an expectation of divorced being more reluctant or hesitating to see a doctor. The former supports an expectation of divorced patients being in worse conditions when hospitalized and more depending on hospital care, given lack of care from a spouse at home.

### Education as ranking variable

Table 5 mirrors Table 4, just with the nine categories educational level used as rank variable instead of income. Likewise, Table 8 Appendix in supplementary materials mirrors Table 7 in Appendix.

**Table 5:** Decomposition of inequality in health care and pharmaceutical usage ranked by education

Ranked by education	SECONDARY CARE										PRIMARY CARE				PHARMACEUTICALS	
	INPATIENT						OUTPATIENT									
	Care		long stays		Rehabilitation		Care		Rehabilitation		General pracitice		Specialist			
Number of observations	318,684		318,684		318,684		209,530		8,089		318,684		318,684		318,684	
Mean	20998.880	***	719.892	***	236.974	***	16830.080	***	3959.272	***	3069.761	***	2108.322	***	7488.065	***
C (observed)	−0.044	***	−0.047	***	−0.064	***	0.006	*	0.027	***	−0.045	***	0.015	***	−0.036	***
C (predicted)	−0.043	***	−0.048	***	−0.062	***	0.005	*	0.028	***	−0.045	***	0.017	***	−0.036	***
C (unexplained)	0.000		0.002		−0.002		0.001		0.000		0.000		−0.002	***	0.000	
Determinants (reference group)	C		C		C		C		C		C		C		C	
SES																
Income	0.06		−1.33		0.28		11.18		0.48		0.48	*	9.62		−0.95	
Educational level (high education)																
Primary education	43.98	***	37.37		7.60		363.57		65.54	*	61.19	***	199.47	***	7.99	
Medium education	−11.41	***	−14.11		2.60		−34.53		−15.47		−15.48	***	−1.73		5.62	**
Age and gender (Males0-14) (Females0-14)																
M15-29	−2.24	***	−3.86	**	0.50	**	34.68		2.29		0.19	***	1.74	***	−0.70	***
M30-44	5.87	***	7.41	*	−1.23	**	−74.69		−4.28		−1.85	***	−3.02	***	2.08	***
M45-59	15.98	***	21.08	**	−6.01	***	−167.99		−13.05		−8.69	***	−2.67		9.86	***
M60-74	20.02	***	29.27	**	−9.48	***	−241.32		−18.19		−14.92	***	5.54	*	15.95	***
M75+	−0.31		−0.37		0.22		−3.22		−4.34		0.27		−0.11		−0.26	
F15-29	−1.81	***	−2.51	*	0.43	**	18.00		1.86		1.22	***	−1.10	***	−1.10	***
F30-44	5.09	***	6.66	*	−1.68	***	−54.11		−4.28		−3.16	***	4.38	***	3.81	***
F45-59	17.68	***	20.85	**	−6.55	***	−180.17		−21.80		−11.47	***	17.85	***	10.23	***
F60-74	24.17	***	30.07	**	−10.89	***	−278.22		−40.58	*	−15.70	***	21.88	***	13.36	***
F75+	−0.38		−0.45		0.23		−3.60		−5.08		0.24		−0.15		−0.21	
Labour market affiliation (in job)																
Not in job (maternity leave, job seeker allowance)	−0.64	***	−1.04	*	−0.51	***	3.47		1.08		−0.52	***	1.12	***	−0.06	
Not in job (unemployment benefit)	0.92	***	0.35		0.33	***	−3.96		−0.84		0.74	***	−1.78	***	0.36	***
Education, training	−0.98	**	−2.83	**	0.52	**	−7.67		1.50		−0.48	***	1.52	**	0.68	*
Early retired	20.88	***	25.70	***	8.21	***	−242.75		1.00		10.30	***	−54.05	***	37.18	***
Retired	26.66	***	29.38	**	13.32	***	−254.03		−12.14		13.41	***	−53.57	***	21.77	***
Child	3.72	***	9.54	*	−2.23	***	16.49		−3.98		−0.78	***	−7.75	***	2.78	***
Marital status (married)																
Unmarried	−0.22		1.04		0.13		2.24		0.02		−0.19	***	1.21	***	0.42	***
widowed/longest living partner	0.96		6.21	**	3.29	***	48.90		6.44		−0.19		13.59	***	0.90	***
Divorced/cancelled partnership	0.90	***	1.96	**	0.99	***	6.64		0.00		0.00		2.07	***	0.96	***
Ethnicity (Ethic Dane)																
Immigrant	6.77	***	7.63	**	4.67	***	−42.78		4.97		−0.78	***	−14.18	***	10.31	***
Descendant	0.10		0.30	*	0.08	*	−0.61		0.31		0.00		−0.26	**	0.15	***
Region of residence (Capital Region of Denmark)																
Region Zealand	0.54	***	−2.35	*	−0.04		6.83		2.77		0.34	***	5.76	***	−0.15	*
Region of Southern Denmark	−2.47	***	−7.99	**	7.77	***	−21.85		47.04	*	2.08	***	19.42	***	−0.77	***
Central Denmark Region	−0.93	***	−4.68	**	3.46	***	13.59		−19.07		1.70	***	8.27	***	−0.37	***
North Denmark Region	−2.22	***	−3.80	**	4.00	***	39.02		6.71		2.99	***	19.12	***	0.16	
Degree of urbanity of residence (cities)																
Suburbs	−0.36	*	0.74		0.04		2.65		−1.93		0.52	***	2.19	***	0.44	***
Country side	−2.21	**	−0.19		−3.92	***	69.52		−22.51	*	4.21	***	16.68	***	0.75	
Morbidity indicators																
Incident in 2011	0.25	***	0.04		0.25	***	3.22		1.63		1.19	***	−5.53	***	1.20	***
Complication group CG1 (CG0) *	0.13		0.15		0.07		8.98		1.05		0.02		−0.09		0.18	
Complication group CG2 (CG0)*	29.52	***	29.86	***	20.87	***	−217.68		−5.92		3.12	***	−19.41	***	14.79	***
Death in 2011	8.81	***	15.21	**	4.37	***	−28.03		−0.08		−0.41	***	2.94	***	−0.78	***

*CG0 = no complications, CG1 = minor complications, CG2 = severe complications

Turning to the regression coefficients (Table 8 in Appendix), some (although minor) differences across regions are found. Thus, the Capital Region and Zealand Region have higher costs for in-patient, out-patient, special care in primary care and pharmaceuticals than the three other regions, whereas the opposite is true for services in general practice. Overall, this pattern is also reflected in the level of urbanity, where especially residents in country side use less resources than patients’ resident in cities, and where costs are more concentrated among patients from higher income levels. This might be explained by the Capital region and cities having more resources to seek up patients and invest in secondary prevention efforts targeting all patients also those belonging to lower SES groups, who might be more difficult to address.

**Fig. 3:**
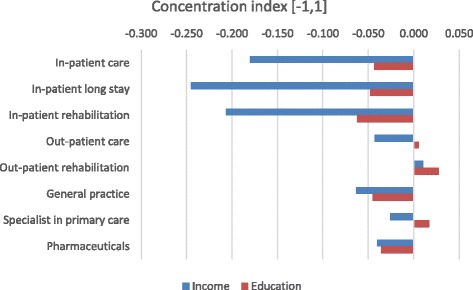
Concentration indices of health care and pharmaceutical usage based on ranking by income and educational level respectively

Concentration indices based on educational versus income ranks are shown in Fig. 3. It is seen that costs for outpatient rehabilitation and specialists in primary care concentrates among the higher socioeconomic groups, but with relatively stronger associations when ranking according to education. This may indicate that educational level is more decisive than income for usage of outpatient services, rehabilitation and specialist in primary care among diabetes patients.

**Fig. 4:**
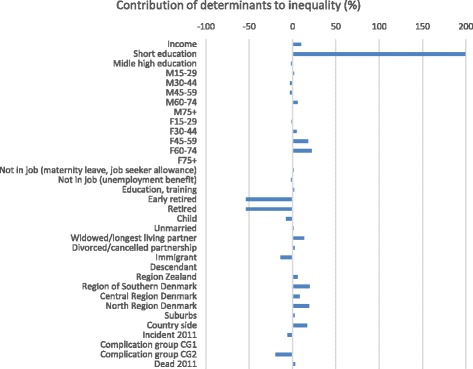
Decomposition of education related inequality in costs for specialists in primary care

Decomposition of inequality of costs for specialists in primary care (Fig. 4) shows that especially for women 45+, residents in the countryside and other regions than the Capital Region, costs concentrate among higher educated patients. For early retired and retired, the opposite pattern is seen with costs concentrating among the lower educated patients, since these patient groups on average have lower educational level than patients in job (as indicated by negative concentration indices in Table 8 in Appendix) and consume more resources since they are more morbid (cf. the positive regression coefficients in Table 8 in Appendix). The same applies for patients with severe complications and for immigrants. For the latter, the explanation is, however, reversed, as immigrants have higher educational level and consume less resources (Table 8 in Appendix). Therefore, their contribution to inequality is in the direction of costs accumulating among the lower educational groups. That immigrants have a higher average educational level than ethnic Danes is counterintuitive. One explanation might be that only the highest educated among immigrants are diagnosed at all, another that the preventive effect of education is not the same among ethnic Danes and immigrants.

Concentrating on ethnicity, the observed pattern for specialist treatment is likewise observed for all three types of in-patient care and pharmaceutical usage. For general practice, the pattern is opposite indicating that immigrants of lower educational levels have higher usage of these services than do ethnic Danes (Table 8 in Appendix).

Turning to morbidity indicators, severe complications and dead in 2011 generally explain more of income inequality than of educational inequality. While between 62 and 97% of income related inequality in costs for in-patient and out-patient care was explained by having severe complications or dying in 2011, the figures are 25–45% for educational level. For out-patient care, as much as 92% of costs accumulating among lower levels of income were explained from these two morbidity indicators. For education on the contrary, as much as 245% of inequality with costs accumulating among higher educated are explained from these two morbidity indicators.

For labor market affiliation, similar patterns are observed across the two tables, however with larger magnitude of contributions of the determinants for income inequality than educational inequality. For age and gender, patterns overall agree across the two tables.

## Discussion

Based on our study of the population of Danish diabetes patients, we demonstrate modest inequality in diabetes patients’ health care and pharmaceutical usage, reflecting that the Danish universal health care system is generally not inequitable. Our results, however, indicate that the amount of inequality explained by patients’ morbidity patterns varies greatly across type of services showing different levels of realized access. This corresponds to previous findings concerning income-related inequality in health care utilization in Denmark [23]. Types of services showing inequality, however, differ from previous findings, suggesting that different utilization patterns are at stake within a specific chronic disease area. This indicates a need of disease specific investigations within the large public health diseases, aiming at informing future strategic efforts and national guidelines. Results indicate that patients of higher SES, especially when SES is measured by educational level, are favored or are more proactive in receiving services when they are seriously ill, and that they likewise are more willing to accept rehabilitation services and seek specialist care, when diagnosed with diabetes. In the Danish health care system, general practice, which is formed by private practice physicians serving under a contract with the government and the regions, serves as gate-keeper with referrals required for specialist treatment [50], which might be perceived as a barrier. Our findings supplement existing literature on differences in especially preventive services and maintenance of life style changes [11]. According to demographic determinants, results point to higher costs among higher income groups among immigrants, elder 75+ years and residents in outer areas. For immigrants, this finding illustrate lower realized access, which along with the findings concerning morbidity in this group stress the importance of reducing the barriers for these patient groups for fully taking advantage of the Danish health care system. Our results demonstrate that confounding is present between income and selected characteristics, in particular age, labor market attachment and diabetes complications, and that attention must be paid to these by properly controlling for them, as is done in the above regression. Also, given the potential endogeneity between income and labor market attachment (in the sense that labor market affiliation is a potential outcome of income), it is recommendable to either substitute or supply with analyses using education rather than income as measure of SES status. However, while lifestyle factors are not accounted for in the study, education may not be a fully true SES measure and thus not “fairly” compared to income as SES.

The present study does not investigate how to change inequality components, however, it does show where the largest potentials for socio-economic related inequality reduction lie, thus providing an important basis for future research and efforts to reduce inequality in diabetes and in health as such. Given marked prevalence increase in diabetes in Denmark as well as globally [21, 51], our findings indicate that inequality in diabetes will also increase. This only stresses the importance of recognition and prioritizing of inequality aspects within chronic disease and diabetes.

The study adds empirical insights regarding choice of proxy for SES. Important differences of applying income and education as proxy for patients’ SES are noted, which may be ascribed to endogeneity of income, i.e., that income, alike other measures of labor market attachment, to a certain extent is determined by morbidity. Our results indicate that education is more decisive than income for patients’ realized access. Hence, education as rank variable might be preferable in analyses of utilization patterns. Application of both education and income, however, may be recommended since the two enables a more nuanced and comprehensive understanding of results. Furthermore, when using education as proxy for SES, several reservations remain as discussed by inter alia Cutler and Lleras-Muney [9] and Cuther et al. (2011). One reservation regards as to whether there is a direct link or merely indirect links via for example lifestyle, which correlates with education. Furthermore, the health effect of an additional year of schooling may be far from constant. Also, the effect of age at educational attainment is pointed out: The lower age at educational attainment, the lower effect of education on health later in life, given that one should expect a diminishing effect of education over the lifespan. The education effect on health may also vary with gender and ethnicity. Finally, unobserved matters like genetic conditions or other intergenerational patterns are pointed out by the authors. For example, children from low SES families may have less educational outcome on health.

There are certain limitations of the study, which should be noticed. Data on patients’ need evaluated from a physician or self-assessed by the patient would enhance the study, as would data on patients’ life style choices. Next, since diabetes is highly influenced by risky health behaviour [36], observed associations might to a certain degree be explained by life style choices [37]. Furthermore, in our regressions, we chose not to include interactions aiming at easing interpretation of results. Also, in the concentration index calculations, tied ranking was applied within each educational level. This might potentially bias results as compared to what would be obtained by a continuous outcome variable. This was investigated by Clarke and Van Ourti [8], who included income level as an approximation for a ranking variable within each of the educational categories. Our results indicate downward as well as upward impacts of applying a grouped rank variable as compared to a continuous variable. Mainly, we find that the grouped variable underestimates the concentration indices. For outpatient care we find that the sign shifts from positive to negative. The concentration index for outpatient care was only significant on a 10% level. The results of Clarke and Van Ourti indicated the largest impact of grouped ranking variable in cases where the concentration index was insignificant. This might indicate that the positive sign for outpatient care can be questioned, however, the positive sign for ambulant rehabilitation remains with all ranking variables. Furthermore, it should be noted that while we use patients’ personal income as a convenient measure of the individual SES, household income (equivalized for household composition) may alternatively be applied as a measure of the full economic capacity of the patient. While the latter summarizes the SES of the household, assuming the SES of the patient to be equal to the SES of the household, the former may be more adequate for more complex household compositions. Anyway, we appreciate that inclusion of household income rather than individual income would more precisely express the economic ability of the patient.

Finally, it should be noted that the methodology behind the study, was developed in the early 2000s [43, 44, 48]. Since then, different authors suggested alternative approaches to address certain aspects. Thus, corrections to the concentration index were suggested by Wagstaff [46] and Errygers [15]. Wagstaff showed that that the upper and lower bounds of a binary variable whose inequality is investigated depend on the mean of this variable, while Errygers showed that this is the case for any variable with bounds. Thus, when a health variable has bounds, the concentration index will depend on the mean, and comparisons between populations with different health means therefore become problematic. Other methods considering that socio-economic inequality is bivariate by nature and measuring the correlation between health and socio-economic status were suggested by Erreygers and Kessels [16], Kessels and Errygers [28], and Erreygers and Kessels [17]. To these, Heckley et al. [24] added a Recentered Influence Function (RIF) regression approach, where a two-dimensional decomposition of determinants of health as well as of determinants of the socio-economic variable (income in the present paper), together with a feed-back between these two, was suggested. Furthermore, the choice of concentration index involves a value judgement as discussed by e.g. Allanson and Paetrie [2] and Kjellson et al. [30]. Thus, a choice must be made between absolute and relative measures, and between measures of health or ill-health in case the index has both a lower and an upper bound. As shown by van Doorslaer and Koolman [42] and Clarke et al. [7], the choice of index can influence the ranking.

## Conclusion

Even in a universal health care system, our results, which are based on the population of Danish diabetes patients, indicate differences in realized access with patients of higher SES, especially higher educational level, to a larger extent enjoying offers of especially out-patient services, rehabilitation and specialists in primary care. Health care usage of patients of lower SES hereby not always corresponds to their need. Especially elder people, divorced, people outside the labour market and immigrants are vulnerable when belonging to lower SES groups and would benefit from being targeted directly. Results indicate that different utilization patterns are at stake within a specific chronic disease as diabetes, compared to general health care utilization patterns. Methodologically, our findings underpin important differences of using income and educational level, respectively, as proxy for SES. Our results indicate that education is more decisive than income for patients’ realized access whereas income-related inequality in health care usage to a higher extent is explained by morbidity. Several of these findings may underpin universal structures behind inequality in diabetes, and in chronic disease in general, which are valuable beyond the specific case of Denmark.

## Appendix

**Table 6:** Grouping of diagnoses and interventions used for classifying hospital activities by complication states of relevance for diabetes, and with respect to diagnostic specificity for diabetes

Diagnosis or procedure	Qualifying for complication state^a^	Specificity for diabetes^b^
Diabetes in pregnancy, childbirth and the puerperium	0	1
Diabetes, without indication of chronic complication	0	1
Hypoglycaemiccoma NOS	0	0
Screening for diabetic retinopathy	0	1
Drug treatment or instruction specific for diabetes	0	1
Acute myocardial infarction	1	0
Diabetes with complication, not further specified	1	1
Diabetes with complications in peripheral vascular system	1	1
Diabetes with eye complication	1	1
Diabetes with footulcer	1	1
Diabetes with microangiopathy	1	1
Diabetes with neurological complication	1	1
Diabetes with peripheral angiopathy	1	1
Diabetes with renal complication	1	1
Diabetic cataract	1	1
Diabetic polyneuropathy	1	1
Diabetic retinopathy not otherwise specified	1	1
Diseases of the lens	1	0
Polyneuropathy	1	0
Proliferative diabetic retinopathy	1	1
Uraemia	1	0
Simplex diabetic retinopathy	1	1
Cataract, retinopathy in diabetes	1	1
Diabetic angiopathy in extremities	1	1
Diabetic nephropathy, Kimmelstiel-Wilson syndrom	1	1
Neuropathy, diabetic polyneuritis, diabetes	1	1
Amputation at or below ankle level	1	0
Coronary bypass operation	1	0
Surgery for eye complication	1	0
Teatment of ulcer of lower limb	1	0
Diabetes with gangraene	2	1
Diabetes with multiple complications	2	1
Diabetic maculopathy	2	1
Dialysis	2	0
Diseases of the retina	2	0
Heart failure	2	0
Kidney transplantation	2	0
Renalfailure	2	0
Stroke	2	0
Blindness	2	0
Diabetic gangraene	2	1
Gangrena of lower limb	2	0
Intracerebral haemorrhage	2	0
Amputation above ankle level	2	0

^a^Value indicates classification state (0, 1 or 2, respectively)

^b^Values 1 and 0 indicate that item is specific for diabetes and unspecific for diabetes, respectively

**Table 7:** Decomposition of inequality in health care costs ranked by income, regression coefficient (B) and concentration index (CI) for each determinant

Ranked by income		SECONDARY CARE										PRIMARY CARE				PHARMACEUTICALS	
		INPATIENT						OUTPATIENT									
		Care		long stays		Rehabilitation		Care		Rehabilitation		General pracitice		Specialist			
Variable (reference group)		Mean		Mean		Mean		Mean		Mean		Mean		Mean		Mean	
Income	b01**	0.00		0.00		0.00		0.00		0.00		0.00		0.00	*	0.00	
	ci01***	0.34	***	0.34	***	0.34	***	0.34	***	0.31	***	0.34	***	0.34	***	0.34	***
Education (high education)																	
low education	b02	1656.98	***	54.93		4.80		−1036.59	***	−287.87	*	349.09	***	−262.41	***	86.52	
	ci02	−0.19	***	−0.19	***	−0.19	***	−0.20	***	−0.19	***	−0.19	***	−0.19	***	−0.19	***
medium education	b03	1025.82	***	44.26		−4.24		−322.94		−137.91		216.14	***	−8.86		−159.67	***
	ci03	0.11	***	0.11	***	0.11	***	0.10	***	0.08	***	0.11	***	0.11	***	0.11	***
Age and gender (Males0-14) (Females0-14)																	
M15-29	b04	−613.27	***	−35.70	***	2.24	**	−450.86	***	−114.50	***	7.93	***	−17.02	***	−59.22	***
	ci04	−0.16	***	−0.16	***	−0.16	***	−0.17	***	−0.14		−0.16	***	−0.16	***	−0.16	***
M30-44	b05	−423.91	***	−17.71	***	1.44	**	−244.19	***	−58.19	***	20.43	***	−7.84	***	−45.28	***
	ci05	0.39	***	0.39	***	0.39	***	0.41	***	0.36	***	0.39	***	0.39	***	0.39	***
M45-59	b06	−283.26	***	−12.50	***	1.75	***	−181.37	***	−20.66		23.57	***	−1.83		−52.54	***
	ci06	0.38	***	0.38	***	0.38	***	0.38	***	0.35	***	0.38	***	0.38	***	0.38	***
M60-74	b07	−227.19	***	−11.16	***	1.79	***	−147.29	***	−15.57		26.02	***	2.06	*	−54.51	***
	ci07	0.02	***	0.02	***	0.02	***	0.01	***	0.03	**	0.02	***	0.02	***	0.02	***
M75+	b08	−228.72	***	−9.51	***	2.72	***	−175.53	***	−26.77	**	30.16	***	2.68	***	−56.25	***
	ci08	−0.21	***	−0.21	***	−0.21	***	−0.19	***	−0.18	***	−0.21	***	−0.21	***	−0.21	***
F15-29	b09	−492.86	***	−22.93	***	1.95	**	−220.61	***	−91.68	***	51.24	***	10.34	***	−90.40	***
	ci09	−0.13	***	−0.13	***	−0.13	***	−0.14	***	−0.07		−0.13	***	−0.13	***	−0.13	***
F30-44	b10	−366.93	***	−16.01	***	1.99	***	−178.72	***	−57.76	***	34.93	***	10.76	***	−82.82	***
	ci10	0.32	***	0.32	***	0.32	***	0.34	***	0.23	***	0.32	***	0.32	***	0.32	***
F45-59	b11	−313.06	***	−12.33	***	1.91	***	−198.61	***	−33.74	**	31.09	***	10.80	***	−54.44	***
	ci11	0.48	***	0.48	***	0.48	***	0.39	***	0.40	***	0.48	***	0.48	***	0.48	***
F60-74	b12	−274.92	***	−11.52	***	2.05	***	−169.68	***	−32.91	***	27.37	***	8.50	***	−45.58	***
	ci12	0.02	***	0.02	***	0.02	***	0.01	***	0.03	**	0.02	***	0.02	***	0.02	***
F75+	b13	−283.56	***	−11.09	***	2.86	***	−196.36	***	−31.08	***	27.64	***	3.61	***	−45.60	***
	ci13	−0.16	***	−0.16	***	−0.16	***	−0.15	***	−0.12	***	−0.16	***	−0.16	***	−0.16	***
Labour market affiliation (in job)																	
Not in job (maternity leave, job seeker allowance)	b14	5656.97	***	310.30	***	76.42	***	2065.49	***	796.35	**	705.00	***	344.42	***	147.77	
	ci14	0.09	***	0.09	***	0.09	***	0.11	***	0.13	***	0.09	***	0.09	***	0.09	***
Not in job (unemployment benefit)	b15	7714.01	***	100.31		45.45	***	1793.17	***	355.39		955.25	***	518.71	***	900.41	***
	ci15	−0.44	***	−0.44	***	−0.44	***	−0.43	***	−0.42	***	−0.44	***	−0.44	***	−0.44	***
Education, training	b16	−3160.16	**	−297.82	***	25.81	***	1328.96	**	−1347.85	**	−227.81	***	−165.51	***	621.87	*
	ci16	−0.90	***	−0.90	***	−0.90	***	−0.91	***	−0.85	***	−0.90	***	−0.90	***	−0.90	***
Early retired	b17	12395.85	***	509.88	***	80.83	***	8147.46	***	−67.01		932.87	***	1113.52	***	6599.71	***
	ci17	−0.15	***	−0.15	***	−0.15	***	−0.14	***	−0.10	***	−0.15	***	−0.15	***	−0.15	***
Retired	b18	7303.15	***	271.48	***	60.31	***	4240.48	***	431.49	*	562.26	***	511.90	***	1784.64	***
	ci18	−0.21	***	−0.21	***	−0.21	***	−0.20	***	−0.18	***	−0.21	***	−0.21	***	−0.21	***
Child	b19	−7903.27	***	−687.25	***	79.26	***	1970.06		−2358.20	*	257.09	***	−591.64	***	−1792.82	***
	ci19	−0.98	***	−0.98	***	−0.98	***	−0.98	***	−0.99	***	−0.98	***	−0.98	***	−0.98	***
Marital status (marriaged)																	
Unmarried	b20	−793.18	*	118.66	**	7.18		−967.62	***	91.72		−98.20	***	−144.60	***	435.56	***
	ci20	0.03	***	0.03	***	0.03	***	0.02	***	0.09	***	0.03	***	0.03	***	0.03	***
widowed/longest living partner	b21	546.13		110.96	**	28.94	***	−1280.55	***	−317.43	*	−14.08		−242.16	***	138.57	***
	ci21	−0.09	***	−0.09	***	−0.09	***	−0.06	***	0.01		−0.09	***	−0.09	***	−0.09	***
Divorced/annuled partnership	b22	1960.90	***	143.55	***	35.08	***	−729.78	**	−89.68		0.94		−154.77	***	625.22	***
	ci22	0.04	***	0.04	***	0.04	***	0.05	***	0.08	***	0.04	***	0.04	***	0.04	***
Ethicity (Ethic Dane)																	
Immigrant	b23	−4598.66	***	−180.16	***	−52.24	***	−1906.88	***	556.50	**	80.42	***	−332.02	***	−2082.04	***
	ci23	−0.21	***	−0.21	***	−0.21	***	−0.22	***	−0.18	***	−0.21	***	−0.21	***	−0.21	***
Descendant	b24	−2601.88		−272.86	***	−32.93	**	−1257.39		1008.95		19.62		−241.26	***	−1152.62	***
	ci24	−0.24	***	−0.24	***	−0.24	***	−0.25	***	−0.01		−0.24	***	−0.24	***	−0.24	***
Region of residence (Capital Region of Denmark)																	
Region Zealand	b25	1518.98	***	−218.02	***	−1.39		−1508.86	***	−1203.57	***	146.81	***	−559.62	***	−121.78	*
	ci25	0.00		0.00		0.00		0.00		−0.03		0.00		0.00		0.00	
Region of Southern Denmark	b26	−2690.03	***	−289.19	***	140.30	***	1281.61	***	−2932.85	***	343.49	***	−731.81	***	−246.01	***
	ci26	−0.04	***	−0.04	***	−0.04	***	−0.03	***	−0.05	***	−0.04	***	−0.04	***	−0.04	***
Central Denmark Region	b27	−2483.33	***	−418.62	***	154.80	***	−1992.48	***	−2226.42	***	697.56	***	−766.99	***	−293.93	***
	ci27	0.00		0.00		0.00		0.00		0.04	***	0.00		0.00		0.00	
North Denmark Region	b28	−3087.03	***	−173.55	***	91.92	***	−3236.22	***	−1330.41	***	632.16	***	−916.56	***	69.90	
	ci28	−0.03	***	−0.03	***	−0.03	***	−0.03	***	0.00		−0.03	***	−0.03	***	−0.03	***
Urbanity (Cities)																	
Suburbs	b29	−750.90	*	52.07		1.25		−439.30		971.95	***	170.91	***	−162.38	***	278.42	***
	ci29	0.00	*	0.00	*	0.00	*	0.00		0.01		0.00	*	0.00	*	0.00	*
Country side	b30	−791.07	**	−1.76		−23.64	***	−1447.17	***	975.13	***	230.68	***	−206.41	***	82.10	*
	ci30	−0.04	***	−0.04	***	−0.04	***	−0.04	***	−0.03	***	−0.04	***	−0.04	***	−0.04	***
Morbidity indicators																	
Incident in 2011	b31	−1287.16	***	−4.70		−21.37	***	1873.66	***	293.34		−949.62	***	−1000.48	***	−1880.24	***
	ci31	0.05	***	0.05	***	0.05	***	0.02	***	0.11	***	0.05	***	0.05	***	0.05	***
Complication group CG1 (CG0) *	b32	8924.98	***	370.12	***	77.74	***	3043.54	***	1104.35	***	158.60	***	192.82	***	3609.95	***
	ci32	0.01	***	0.01	***	0.01	***	0.04	***	0.05	***	0.01	***	0.01	***	0.01	***
Complication group CG2 (CG0)*	b33	29216.04	***	993.73	***	342.16	***	11567.27	***	2064.34	***	469.02	***	662.58	***	4359.56	***
	ci33	−0.13	***	−0.13	***	−0.13	***	−0.10	***	−0.07	***	−0.13	***	−0.13	***	−0.13	***
Death in 2011	b34	87725.75	***	5083.04	***	722.43	***	8603.19	***	−912.07	***	−617.85	***	−1014.66	***	−2310.90	***
	ci34	−0.68	***	−0.68	***	−0.68	***	−0.63	***	−0.53	***	−0.68	***	−0.68	***	−0.68	***

*CG0 = no complications, CG1 = minor complications, CG2 = severe complications

** b*N =* Regression coefficient of variable N

***ci*N =* Concentration index of variable N

**Table 8:** Decomposition of inequality in health care costs ranked by education, regression coefficient (B) and concentration index (CI) for each determinant

Ranked by education		SECONDARY CARE										PRIMARY CARE				PHARMACEUTICALS	
		INPATIENT						OUTPATIENT									
		Care		long stays		Rehabilitation		Care		Rehabilitation		General pracitice		Specialist			
Variable (reference group)		Mean		Mean		Mean		Mean		Mean		Mean		Mean		Mean	
Income	b01	0.00		0.00		0.00		0.00		0.00		0.00	*	0.00	*	0.00	
	ci01	0.11	***	0.11	***	0.11	***	0.11	***	0.10	***	0.11	***	0.11	***	0.11	***
Education (high education)																	
low education	b02	1671.70	***	57.60		5.09		−1044.36	***	−302.94	*	349.05	***	−260.11	***	91.10	
	ci02	−0.58	***	−0.58	***	−0.58	***	−0.58	***	−0.57	***	−0.58	***	−0.58	***	−0.58	***
medium education	b03	1055.65	***	47.62		−3.79		−334.48		−145.09		215.94	***	−6.32		−152.72	**
	ci03	0.25	***	0.25	***	0.25	***	0.25	***	0.28	***	0.25	***	0.25	***	0.25	***
Age and gender (Males0-14) (Females0-14)																	
M15-29	b04	−620.08	***	−35.51	***	2.27	**	−454.78	***	−112.13	***	7.99	***	−16.68	***	−58.14	***
	ci04	−0.14	***	−0.14	***	−0.14	***	−0.13	***	−0.16	*	−0.14	***	−0.14	***	−0.14	***
M30-44	b05	−426.18	***	−17.70	***	1.47	**	−247.64	***	−56.56	***	20.41	***	−7.58	***	−45.05	***
	ci05	0.08	***	0.08	***	0.08	***	0.10	***	0.08	**	0.08	***	0.08	***	0.08	***
M45-59	b06	−284.55	***	−12.48	***	1.77	***	−183.38	***	−19.55		23.57	***	−1.65		−52.41	***
	ci06	0.07	***	0.07	***	0.07	***	0.07	***	0.10	***	0.07	***	0.07	***	0.07	***
M60-74	b07	−229.27	***	−11.19	***	1.80	***	−149.42	***	−14.46		26.00	***	2.20	*	−54.49	***
	ci07	0.05	***	0.05	***	0.05	***	0.06	***	0.07	***	0.05	***	0.05	***	0.05	***
M75+	b08	−229.99	***	−9.48	***	2.74	***	−177.51	***	−25.93	**	30.16	***	2.81	***	−56.25	***
	ci08	0.00		0.00		0.00		0.00		0.03		0.00		0.00		0.00	
F15-29	b09	−500.86	***	−23.04	***	1.98	**	−224.03	***	−88.97	***	51.19	***	10.54	***	−90.45	***
	ci09	−0.11	***	−0.11	***	−0.11	***	−0.11	***	−0.08	*	−0.11	***	−0.11	***	−0.11	***
F30-44	b10	−369.02	***	−15.98	***	2.02	***	−181.68	***	−56.30	***	34.92	***	10.99	***	−82.49	***
	ci10	0.07	***	0.07	***	0.07	***	0.08	***	0.05	**	0.07	***	0.07	***	0.07	***
F45-59	b11	−314.80	***	−12.28	***	1.93	***	−200.69	***	−32.70	**	31.10	***	10.98	***	−54.35	***
	ci11	0.09	***	0.09	***	0.09	***	0.08	***	0.11	***	0.09	***	0.09	***	0.09	***
F60-74	b12	−276.81	***	−11.50	***	2.07	***	−171.59	***	−31.94	**	27.36	***	8.67	***	−45.62	***
	ci12	0.07	***	0.07	***	0.07	***	0.06	***	0.08	***	0.07	***	0.07	***	0.07	***
F75+	b13	−284.82	***	−11.08	***	2.87	***	−198.21	***	−30.10	***	27.64	***	3.74	***	−45.61	***
	ci13	0.00		0.00		0.00		0.00		0.02		0.00		0.00		0.00	
Labour market affiliation (in job)																	
Not injob (maternity leave, job seeker allowance)	b14	5647.67	***	310.49	***	75.85	***	2050.64	***	801.14	*	705.60	***	343.97	***	146.35	
	ci14	0.05	***	0.05	***	0.05	***	0.05	***	0.04		0.05	***	0.05	***	0.05	***
Not in job (unemployment benefit)	b15	7754.26	***	102.21		45.48	***	1755.88	***	345.08		954.64	***	520.03	***	905.78	***
	ci15	−0.05	***	−0.05	***	−0.05	***	−0.06	***	−0.11	**	−0.05	***	−0.05	***	−0.05	***
Education, training	b16	−3047.98	**	−296.37	***	26.77	***	1239.85	**	−1327.45	**	−226.53	***	−163.62	***	634.89	**
	ci16	−0.36	***	−0.36	***	−0.36	***	−0.38	***	−0.27	***	−0.36	***	−0.36	***	−0.36	***
Early pensioner	b17	12424.47	***	516.77	***	80.95	***	8113.88	***	−61.04		932.31	***	1111.59	***	6598.69	***
	ci17	−0.13	***	−0.13	***	−0.13	***	−0.13	***	−0.12	***	−0.13	***	−0.13	***	−0.13	***
Pensioner	b18	7360.21	***	273.17	***	60.90	***	4243.02	***	430.83	*	563.12	***	510.89	***	1791.89	***
	ci18	−0.06	***	−0.06	***	−0.06	***	−0.06	***	−0.05	***	−0.06	***	−0.06	***	−0.06	***
Child	b19	−8016.24	***	−679.84	***	79.79	***	1863.83		−2312.99	*	257.14	***	−578.69	***	−1788.10	***
	ci19	0.76	***	0.76	***	0.76	***	0.75	***	0.66	***	0.76	***	0.76	***	0.76	***
Marital status (marriaged)																	
Unmarried	b20	−762.99	*	119.34	**	7.51		−983.54	***	87.62		−98.00	***	−144.59	***	436.33	***
	ci20	−0.02	***	−0.02	***	−0.02	***	−0.01	***	0.00		−0.02	***	−0.02	***	−0.02	***
widowed/longest living partner	b21	498.17		111.25	**	28.21	***	−1264.30	***	−328.09	**	−14.91		−243.26	***	139.54	***
	ci21	−0.11	***	−0.11	***	−0.11	***	−0.11	***	−0.12	***	−0.11	***	−0.11	***	−0.11	***
Divorced/annuled partnership	b22	1959.70	***	144.38	***	35.48	***	−722.93	**	−93.74		0.99		−155.19	***	624.10	***
	ci22	−0.03	***	−0.03	***	−0.03	***	−0.03	***	0.00		−0.03	***	−0.03	***	−0.03	***
Ethicity (Ethic Dane)																	
Immigrant	b23	−4583.20	***	−184.46	***	−52.36	***	−1895.54	***	566.97	**	80.27	***	−331.87	***	−2081.26	***
	ci23	0.15	***	0.15	***	0.15	***	0.14	***	0.11	***	0.15	***	0.15	***	0.15	***
Descendant	b24	−2549.32		−270.53	***	−33.09	**	−1229.19		1017.49		19.23		−236.49	***	−1134.00	***
	ci24	0.11	***	0.11	***	0.11	***	0.12	***	0.13		0.11	***	0.11	***	0.11	***
Region of residence (Capital Region of Denmark)																	
Region Zealand	b25	1529.58	***	−219.20	***	−1.67		−1514.75	***	−1215.46	***	146.88	***	−560.24	***	−125.67	*
	ci25	−0.02	***	−0.02	***	−0.02	***	−0.02	***	−0.02		−0.02	***	−0.02	***	−0.02	***
Region of Southern Denmark	b26	−2687.59	***	−291.85	***	140.25	***	1267.48	***	−2931.74	***	344.33	***	−731.61	***	−251.59	***
	ci26	−0.03	***	−0.03	***	−0.03	***	−0.03	***	−0.04	***	−0.03	***	−0.03	***	−0.03	***
Central Denmark Region	b27	−2496.60	***	−422.31	***	154.16	***	−2012.19	***	−2215.80	***	696.83	***	−767.81	***	−298.20	***
	ci27	−0.01	***	−0.01	***	−0.01	***	−0.02	***	0.03	***	−0.01	***	−0.01	***	−0.01	***
North Denmark Region	b28	−3077.69	***	−177.70	***	91.88	***	−3251.57	***	−1313.66	***	631.06	***	−916.52	***	63.75	
	ci28	−0.06	***	−0.06	***	−0.06	***	−0.06	***	−0.06	***	−0.06	***	−0.06	***	−0.06	***
Urbanity (Cities)																	
Suburbs	b29	−769.59	*	52.32		1.32		−421.47		975.00	***	171.33	***	−162.57	***	278.79	***
	ci29	−0.03	***	−0.03	***	−0.03	***	−0.03	***	−0.01		−0.03	***	−0.03	***	−0.03	***
Country side	b30	−794.58	**	−1.55		−23.33	***	−1437.32	***	979.44	***	229.74	***	−206.84	***	79.69	
	ci30	−0.06	***	−0.06	***	−0.06	***	−0.06	***	−0.05	***	−0.06	***	−0.06	***	−0.06	***
Morbidity indicators																	
Incident in 2011	b31	−1291.89	***	−5.98		−21.65	***	1862.19	***	307.23		−950.00	***	−999.99	***	−1878.38	***
	ci31	0.02	***	0.02	***	0.02	***	0.02	***	0.07	***	0.02	***	0.02	***	0.02	***
Complication group CG1 (CG0) *	b32	8970.85	***	371.90	***	78.17	***	3050.01	***	1100.95	***	159.51	***	193.67	***	3614.41	***
	ci32	0.00		0.00		0.00		0.01	***	0.00		0.00		0.00		0.00	
Complication group CG2 (CG0)*	b33	29218.30	***	994.70	***	342.29	***	11571.10	***	2057.79	***	469.32	***	662.94	***	4362.94	***
	ci33	−0.03	***	−0.03	***	−0.03	***	−0.03	***	−0.01		−0.03	***	−0.03	***	−0.03	***
Death in 2011	b34	87694.00	***	5094.08	***	720.90	***	8658.61	***	−921.76	***	−618.34	***	−1012.35	***	−2311.31	***
	ci34	−0.02	***	−0.02	***	−0.02	***	−0.05	***	0.00		−0.02	***	−0.02	***	−0.02	***

*CG0 = no complications, CG1 = minor complications, CG2 = severe complications

** b*N =* Regression coefficient of variable N

***ci*N =* Concentration index of variable N

## Abbreviations

C: Concentration Index
CG0: Complication Group 0
CG1: Complication Group 1
CG2: Complication Group 2
DD2: Diabetes Database 2
DRG: Diagnosis Related Grouping
NDR: Danish National Diabetes Register
SD: Statistics Denmark
SES: Socio-Economic Status
WHO: World Health Organization
